# WADD-SEPD Consensus on Psychological Treatment of Dual Disorders I: General Recommendations, Most Used Therapies, and Severe Mental Disorders

**DOI:** 10.3390/jcm15020730

**Published:** 2026-01-16

**Authors:** Ana Benito, Susana Jiménez-Murcia, Judit Tirado-Muñoz, Ana Adan

**Affiliations:** 1 World Association on Dual Disorders (WADD), 28232 Madrid, Spain; judit.tirado@universidadeuropea.es (J.T.-M.); aadan@ub.edu (A.A.); 2 Spanish Society of Dual Disorders (SEPD), 28012 Madrid, Spain; sjimenez@bellvitgehospital.cat; 3 TXP Research Group, Medicine Department, Universidad Cardenal Herrera-CEU, CEU Universities, 12006 Castellón, Spain; 4 Torrente Mental Health Unit, General University Hospital of Valencia, 46900 Valencia, Spain; 5 Clinical Psychology Department, Bellvitge University Hospital-IDIBELL, 08907 Barcelona, Spain; 6 School of Medicine and Health Sciences, University of Barcelona, 08036 Barcelona, Spain; 7 Physiopathology of Obesity and Nutrition, CIBERobn, ISCIII, 28029 Barcelona, Spain; 8 Department of Psychology, Faculty of Biomedical and Health Sciences, Universidad Europea de Madrid, 28670 Madrid, Spain; 9 Network of Research in Primary Care of Addictions (RIAPAD), Carlos III Health Institute, 28029 Madrid, Spain; 10 Department of Clinical Psychology and Psychobiology, University of Barcelona, 08035 Barcelona, Spain; 11 Institute of Neurosciences, University of Barcelona, 08035 Barcelona, Spain

**Keywords:** dual disorders, dual diagnosis, severe mental disorders, schizophrenia, bipolar disorder, depressive disorders, obsessive compulsive disorder, psychological treatment, psychotherapy, consensus

## Abstract

**Background/Objectives**: The treatment of dual disorders (DDs) must be comprehensive and multidisciplinary. There is evidence supporting the effectiveness of psychotherapy in their treatment. However, clinical guidelines, consensus statements, and reviews on the treatment of DDs typically devote considerably less space to psychological therapy than to pharmacological therapy. Therefore, this work aimed to synthesize the available evidence, recommendations, and clinical experience on the psychological treatment of DDs to reach a consensus. **Methods**: Two consensus methods were sequentially implemented: the nominal group technique and the Delphi method. **Results:** The first part of this consensus review encompassed a compilation of general recommendations for the psychological treatment of DDs, evidence on the efficacy of the most frequently used therapies, and recommendations for the psychological treatment of severe dual mental disorders. These disorders include schizophrenia and other psychotic disorders, bipolar disorders, depressive disorders, and obsessive compulsive disorders. **Conclusions**: (1) Psychological treatment is effective; (2) integrated psychological treatment is more effective; (3) motivational interviewing, cognitive behavioral therapy, and relapse prevention are the psychological interventions with the most supporting evidence; (4) the best alternative is multicomponent strategies; (5) the most frequently studied severe mental disorders are schizophrenia and depression; (6) for dual schizophrenia, motivational interviewing and integrated cognitive behavioral therapy combined with other components are recommended; (7) for dual depression, cognitive behavioral therapy with relapse prevention or motivational interviewing is recommended; (8) for dual bipolar disorder, group therapies with psychoeducation or relapse prevention and inclusion of the family, contingency management, and family intervention are recommended; (9) more empirical evidence is needed, especially for obsessive compulsive and schizoaffective disorders; and (10) more randomized clinical trials are needed to improve current methodological limitations.

## 1. Introduction

The term ‘dual disorder’ (DD) refers to the concurrent presence of a substance use disorder (SUD) and another mental disorder (MD) [1] and indicates individuals who suffer from both an addiction and another MD, either concurrently or in sequential order [2]. DD is a different and broader term than ‘comorbidity’, which refers to the temporary coexistence of two or more psychiatric disorders [2].

DDs are present in 25.8% of individuals with an MD, 36.5% of those with an SUD, and 17.8% of those with either disorder [3]. In the case of severe mental disorder (SMD), 28–75% present with DD [4,5]. In children and adolescents receiving treatment for MDs, presence of DD ranges from 18.3% to 54% [6], and 64–88% of adolescents with SUD have at least one other MD [1]. According to data from the Substance Abuse and Mental Health Services Administration [4], the odds ratios of individuals with SUDs experiencing major depression, bipolar disorder (BD), antisocial personality disorder (APD), borderline personality disorder (BPD), panic disorder, specific phobia, or generalized anxiety disorder range from 1.2 to 6.4, reaching 9 in the case of people with multiple MDs. In addition, rates of MD appear to increase as the number of SUDs increases; individuals with polydrug use (more than two substances) are particularly vulnerable to DDs.

DDs are associated with a higher likelihood of hospitalization [4], greater functional impact, lower treatment adherence [7], and increased healthcare costs, morbidity, and mortality, complicating treatment outcomes and prognosis [1,8], with greater severity of addiction and psychiatric symptoms, and suicidal ideation and behavior [9]. Furthermore, DDs are strongly associated with unemployment, homelessness, incarceration, or involvement in the criminal justice system [4]. Despite this, DDs are underestimated, underdiagnosed, and often undertreated [10]. It is estimated that more than 90% of adults with DDs do not receive treatment for both disorders [4].

DD treatment requires a comprehensive approach with multidisciplinary teams [11], and there is evidence of the effectiveness of psychotherapy in this treatment [12]. However, clinical guidelines, consensus statements, and reviews on DD treatment [13], based on a combination of expert opinion, clinical experience, and research evidence [14], generally devote disproportionately limited attention to psychological therapy than to pharmacological therapy. Because psychotherapy is the treatment of choice in most SUDs and is recommended in all SUDs and MDS, it is necessary to have evidence-based information for use in DDs. Therefore, this work aimed to synthesize evidence, recommendations, and clinical experience on the psychological treatment of DDs into a unified consensus for the first time.

The novel aspects of this consensus are: a specific focus on psychological treatment; integration of existing evidence; general recommendations for the treatment of dual disorders, specific to each combination of disorders, and specific according to severity; synthesis of the conclusions that can be reached from the existing evidence; highlighting the gaps in knowledge; and making proposals for future research.

## 2. Materials and Methods

Two consensus methods were sequentially implemented: the nominal group technique and the Delphi method [15]. A group of four psychologists specialized in DD was formed and has been meeting annually since 2022 within the framework of the National Congress of the Spanish Society of Dual Disorders (SEPD) and the International Congress of the World Association on Dual Disorders (WADD) to review, share, and discuss the available empirical evidence, recommendations, and clinical experiences concerning the psychological treatment of DDs. The four members of the group were women. Each year, the group invited three more psychologists who were experts in dual disorders. In total, fifteen other psychologists participated in the meetings: twelve women and three men. The experts included academics, researchers, and clinicians. Most of them worked in Europe and South America. Prior to each in-person meeting, each expert conducted a bibliographic search using the following keywords: (“dual disorders” OR “dual pathology” OR “comorbidity”) AND (“psychological treatment” OR “psychotherapy”). The PubMed, Cochrane, Scopus, Web of Science, PsycINFO, and PubPsych databases were used, without filters. Each expert selected the data they considered most relevant using the following criteria: (1) data on the psychological treatment of DDs, (2) based on empirical evidence, and (3) extracted from peer-reviewed works. The results were shared at the meeting, and conference attendees were also given the opportunity to participate in the debate. The results obtained from the four face-to-face meetings, in conjunction with a new bibliographic search updated in September 2025, were used to draft a preliminary consensus document. All data and evidence included had to have the unanimous consensus of the four authors. This document was subsequently reviewed by the authors in successive versions until the final content was approved. If there had been any disagreement, that content would not have been included in the consensus, although this did not happen.

This first part of the consensus includes the following sections: Recommendations for the psychological treatment of DDs (applicable to all possible comorbidities between SUD and other MDs); evidence on the most used psychological therapies; and recommendations for the psychological treatment of specific dual SMDs (applicable to the SMDs listed in the order in which they appear in the DSM-5-TR [16] and, within each disorder, those applicable to all SUDs followed by those applicable to each specific SUD, also in the order in which they appear in the DSM-5-TR) [16]. For each of the specified SMDs, if the section referring to their comorbidity with a specific SUD does not appear, it means that no literature on the subject has been found.

The level of evidence rating (when available) was documented in parentheses. These ratings are interpreted as follows [17]: 1—Well-designed, randomized, controlled clinical trials or meta-analyses; 2—Clinical trials with minor methodological limitations; 3—Descriptive, comparative, and case–control studies; and 4—Consensus of expert committees, technical reports from health authorities, and case series. The grade of the recommendations, when available—also in parentheses—was interpreted as A—Maximum, B—High, C—Medium, D—Low [17,18]; or Strong and Weak [19]. When no level or grade is specified, it should be considered as level 4, grade D, and a weak recommendation.

## 3. Results

### 3.1. Recommendations for the Psychological Treatment of Dual Disorders

Psychological treatment must be part of a comprehensive treatment provided by a multidisciplinary team, given the multifactorial nature of DDs [11]. The most recommended treatment is an integrated approach [1,12,20] (the same team of professionals manages both disorders within the same setting) [21] with a stepped care approach. That is, a single treatment plan that encompasses both mental health and addiction, with two fundamental objectives: improving access to care by providing individualized treatment and combining psychopharmacological resources and psychosocial tools in a coherent manner [1,4]. This stepped care model [22] has the following stages [23]: Engagement (building the therapeutic alliance), Persuasion (psychoeducational and motivational strategies), Active Treatment (the patient actively participates by collaborating in goal setting), and Relapse Prevention (RP).

Psychosocial interventions play a pivotal role in DDs and ought to be initiated early in treatment. These interventions should be both intensive and built on established interventions for the treatment of MD and substance use, with suitable modifications [24]. Following the stabilization of the acute phase of MD, non-pharmacological treatment assumes a central role, as evidenced by the documented beneficial effects of psychosocial approaches on maintaining abstinence, medication adherence, maintaining a healthy lifestyle, improving community integration, job rehabilitation, and overall improved functioning [21]. Psychosocial and motivational interventions have been shown to be effective in enhancing emotional functioning and preventing relapses, thereby increasing long-term adherence to recovery plans [25,26,27].

Within psychosocial treatments, psychological therapies are a central component. The establishment of an adequate therapeutic relationship is essential in all psychological treatments, and this is even more important in the case of DDs [14]. A quality therapeutic relationship founded upon mutual respect constitutes an indispensable component of effective treatment for individuals diagnosed with DDs [22]. Consequently, the use of rigid rules and confrontational strategies is discouraged [28,29]. Empathy, respect, and trust in the individual’s resilience are considered fundamental attitudes and values for therapists [22,29]. Furthermore, effective management of countertransference is imperative for the establishment of a good therapeutic relationship [4]: being attentive to strong personal reactions and prejudices toward people with DDs; obtaining increased supervision when countertransference is suspected and may be interfering with therapy; and receiving formal and periodic clinical supervision.

The utilization of the Stages of Change Model [30] is encouraged to guide interventions in the psychological treatment of DDs, basing management and the selection of interventions on the patient’s readiness for change. This may differ for SUDs and MDs [22,31].

A range of psychological treatment approaches is employed in DDs, with varying levels of empirical evidence supporting their efficacy. These have been delivered individually or in groups [21], primarily in person, although delivery via eHealth or telehealth has expanded since the onset of the COVID-19 pandemic [14]. Furthermore, it is recommended to adapt treatment to the needs of specific populations, including cultural and linguistic diversity, gender and sexual diversity, age, homelessness, sex work, penal/forensic system, coerced/compelled treatment, and cognitive limitations [4,14,22].

### 3.2. Evidence on the Effectiveness of Psychological Treatments in Dual Disorders

The limited number of randomized controlled trials on psychological interventions for DDs impacts the availability of evidence-based support for various treatment options [22]. This paucity of evidence partly reflects the systematic exclusion of people with SUD from clinical trials for MDs [14]. Consequently, the evidence supporting the psychological treatment of comorbidity between SUD and MDs is considerably more restricted than that for both disorders when considered separately [19]. It is also due to the significant heterogeneity of studies regarding sample characteristics, settings, type of substance used (frequently without specification), type of MD, type of psychological intervention delivered, adherence levels, definitions of standard care, and the intervention outcomes considered [19,21]. All of this makes it difficult to compare the results obtained from different studies and draw conclusions [19].

Psychological interventions are evaluated in smaller-scale studies with greater methodological limitations than pharmacological interventions, often lacking a control group and with small sample sizes and high experimental mortality [20]. Moreover, the proliferation of multicomponent therapies, the absence of delineation of the clinical characteristics of participants during the intervention, the high rate of attrition, and the lack of experimental control in some studies compromise the possibility of considering the current recommended intervention guidelines as well-established treatments [20]. Therefore, meta-analyses conclude that there is insufficient evidence to support the effectiveness of any psychosocial intervention over another or over standard treatment [21]. Nevertheless, brief interventions do appear to be beneficial and should be offered whenever possible. The most frequently utilized brief interventions are printed information, brief counseling/advice sessions, motivational interviewing (MI) [32], brief counseling, questionnaires and self-assessments, self-help manuals, consumption control programs, and videos [22].

The dearth of empirical evidence is particularly pronounced in specific areas of study, for instance, in dual cannabis use disorder [33] and dual behavioral addictions [34]. Moreover, research on children and adolescents with DDs is particularly limited [35].

Taking these limitations into account, we describe the evidence and recommendations for each of the most used psychological therapies for DDs (listed in alphabetical order). The summary of the evidence found and the consensus recommendations can be seen in Table 1.

#### 3.2.1. Acceptance and Commitment Therapy (ACT)

Although evidence is limited, ACT [36] improves abstinence in people with mood disorders and alcohol use disorders [14] and is effective for patients with SUD and PTSD (level of evidence 3) [17]. Furthermore, it has been successfully adapted to digital health interventions [14].

#### 3.2.2. Behavioral Activation (BA)

No evidence has been found regarding the use of BA in DDs in general, but it may be useful in dual depression [14] and for RP.

#### 3.2.3. Cognitive Behavioral Therapy (CBT)

CBT techniques are employed in the active phase of treatment and in post-treatment rehabilitation [34,37], with demonstrated efficacy in the treatment of depression [22,25].

CBT (alone or in combination with pharmacological treatment) is effective in the treatment of dual depressive disorder, although it appears that some improvement is lost after treatment ends (level of evidence 2) [17]. It is also effective for addressing patients with SUD and PTSD (level of evidence 3), in the treatment of adolescents with SUD and psychopathological problems to reduce substance use and improve family relationships in the short term (level of evidence 2), and in the treatment of adolescents with alcohol use problems and suicidal tendencies (level of evidence 2) [17]. However, for comorbidity with SMD, CBT alone shows little consistent support [38]. In a recent meta-analysis, Magill et al. [39] found that integrated CBT showed greater benefits than usual treatment and control comparators with respect to substance use, but not with respect to psychosocial outcomes, suggesting modest efficacy.

Cognitive enhancement [40] is a promising treatment studied for dual stimulant use disorder, but further research is needed.

#### 3.2.4. Contingency Management (CM)

Although CM is promising [22], very few studies have evaluated it [38]. It has broad support in the United States, with studies indicating improvements in treatment retention, drug use, and therapy attendance. However, it has limited support or application in other countries [22].

CM tends to be narrowly focused on substance use, but the results appear to generalize to other data of interest [41]. It shows consistent positive effects on SUD, although studies are limited by the heterogeneity of interventions, participants, methods, outcomes, and measures [41]. Studies have also shown its effectiveness in reducing substance use and psychiatric symptoms in people who use stimulants and have SMD [14]. In this regard, there are contingency programs that have proven useful [34,37], such as the Behavioral Treatment for Substance Abuse in Severe and Persistent Mental Illness (BTSAS) [42].

#### 3.2.5. Dialectical Behavior Therapy (DBT)

DBT [43] is effective in treating individuals with SUD and BPD (level of evidence 3) [17], personality disorders (PDs) (including those co-occurring with SUDs), and SUDs (including those co-occurring with PDs). Recent research supports its use for eating disorders co-occurring with SUDs [14]. Furthermore, among individuals with PD, it has been shown to be equally effective when administered in person via videoconference [44].

#### 3.2.6. Exposure Therapy

Exposure Therapy can be used to successfully treat anxiety disorders and PTSD that co-occur with SUDs. It has been used to reduce relapses and cravings among individuals with alcohol dependence and to mitigate cravings in those using cannabis, methamphetamines, and opiates [14]. However, this evidence is contradictory across studies and limited by methodological factors.

#### 3.2.7. Family or Family-Based Therapy (FT)

There is evidence supporting the effectiveness of family interventions as part of DD treatment [22]. Family groups can be a space for education and support, as family intervention integrates essential information, guidance, and support, while enabling discreet engagement and continuous evaluation [29,31].

Family involvement in the assessment, planning, and delivery of care for DD patients is crucial for their collaboration and support, and ultimately, for the final outcome of treatment [22]. In certain cases, some families may require intensive FT [29]. The benefits of FT include strengthening relationships, the ability to express feelings and thoughts in a safe environment and learning how to support patients during stressful times while reinforcing adaptive coping behaviors [25].

#### 3.2.8. Group Therapy (GT)

All the therapies described in this section can be administered in a group setting. In addition, there are interventions specifically designed for group settings.

Group interventions have proven effective in increasing treatment participation and abstinence rates, as well as reducing the need for hospitalization [29]. GT is widely and effectively used with patients with SUDs, including those diagnosed with schizophrenia. A variety of approaches can be employed, including psychoeducational, medication management, twelve-step, educational, supportive, and social skills enhancement [29].

Referral of patients with SUDs to self-help groups (such as Alcoholics Anonymous, Narcotics Anonymous, SMART Recovery, or others) is common [45]. In many cases, patients who attend Alcoholics Anonymous have higher rates of alcohol abstinence compared to those who do not [45]. While the research is not conclusive, attending traditional twelve-step groups may be beneficial for some patients with DDs, but patients with SMD may encounter difficulties attending these groups [29].

Group counseling has shown consistent positive effects for SMD and SUD, although studies are limited by the heterogeneity of interventions, participants, methods, outcomes, and measures [41]. GT may require modifications and patients with DDs should be supplemented with individual therapy sessions [29].

Dual diagnosis groups (such as Dual Diagnosis Anonymous or Dual Recovery Anonymous) are a specific option of interest. Since all attendees are affected by MDs, they are less prone to misunderstandings and contradictory messages about their psychiatric symptoms or the use of psychotropic medications, a phenomenon that sometimes arises in conventional twelve-step groups [29].

#### 3.2.9. Interpersonal Therapy

Interpersonal therapy [46] has been adapted for patients with cocaine and/or opioid dependence, although there is limited evidence of its use in people with DDs [22].

#### 3.2.10. Mindfulness (MF)

MF can be helpful for people with DDs, who often maintain a cycle of mental illness and substance use, as it helps develop greater awareness of automatic thought patterns [14]. It has been incorporated into the Mindfulness-Based Relapse Prevention program [47], which is an eight-week group program.

#### 3.2.11. Motivational Interviewing (MI)

MI is the most strongly evidence-based psychological intervention, being effective in the treatment of DDs [22,25]. MI improves behavior change, treatment participation, attendance, and retention, while increasing motivation and confidence among patients with DD [4]. Motivational interventions reduce substance use and improve psychopathological symptoms, particularly among populations experiencing extreme hardship, such as the homeless and active injectors with no intention of quitting (Grade B recommendation) [17,34]. MI is particularly useful in helping patients assess their perception of their problems, their understanding of their disorders, and their desire to continue treatment. Furthermore, it improves attendance at initial sessions and increases willingness to take responsibility for change [4]. Therefore, MI is an important intervention for increasing adherence to treatment, including pharmacological treatment [48].

MI has shown better results than psychoeducational interventions [34,37], and there is considerable evidence supporting its use in treating MDs and alcohol use disorders [14]. Though there are some inconsistencies, the results show that MI has the highest quality evidence for reducing substance use in the short term for SMDs and SUDs [38], but more research is needed [4]. Motivational interventions can be used to treat consumers at all stages of treatment [34,37] and can also be applied to motivate change regarding mental health conditions [14]. In fact, a modified version of traditional MI has been used for patients with mental health conditions and SUDs [22].

#### 3.2.12. Psychoeducation (PE)

PE is an important part of the care plan for people with DDs. It provides patients and their families with information to help them understand and better cope with substance use and mental health issues. This involves strengthening their resources and coping skills [22,25,48].

#### 3.2.13. Relapse Prevention (RP)

Research supports the use of RP for improving substance use outcomes in people with DD [4]. RP is effective in reducing the severity of PTSD symptoms, as well as the amount, frequency, and severity of substance use in people with PTSD and SUD [14].

Several RP interventions have been developed for patients with DDs [29]. Tailored RP therapy promotes adherence to treatment, including medication, which is crucial for people with psychotic disorders or BD. It also improves social functioning and helps patients meet their basic needs, such as finding housing or stable employment [4]. RP can be delivered individually or in small groups and may include practice or role-playing to learn how to effectively cope with high-risk situations [4].

#### 3.2.14. Multicomponent Therapies

Rather than applying isolated therapies, a combination approach is recommended in DDs. That is, therapies comprising several active components that, together, attempt to address all possible aspects required by the intervention. However, this approach has a key methodological limitation: it is impossible to know which components are responsible for the demonstrated efficacy [20].

Improvements in substance use, mental state, and treatment adherence have been observed when MI is combined with CBT [34,37,38]. Although no single treatment has proven superior, this combination is considered the most successful intervention [27]. A combination of CBT principles (e.g., self-regulation skills and cognitive restructuring), MI, and MF is also recommended to help develop self-regulation skills, MF, flexibility, and cognitive reappraisal [12].

Better results are obtained by combining MI with positive reinforcement, social skills development, and GT, always within the context of a proactive approach and with interventions adapted to each stage [1]. Motivational reinforcement, simple CBT-based strategies, relaxation techniques, and grounding techniques can be useful for controlling substance use and MDs [14]. Furthermore, combining MI, CBT, and family interventions improves outcomes for patients with SMDs and SUDs [34,37].

**Table 1:** Summary of evidence on the effectiveness of different psychological therapies in the treatment of dual disorders.

Therapy	Evidence in Dual Disorders	Evidence in Specific Disorders and Uses
**Acceptance and** **Commitment Therapy**	Limited evidence.	Mood disorders and alcohol consumption. Dual post-traumatic stress disorder. Digital health interventions.
**Behavioral activation**	There is no evidence.	Dual depression. Relapse prevention.
**Cognitive behavioral therapy**	Effective, supported by extensive evidence for active treatment and relapse prevention.	Dual depressive disorder. Dual post-traumatic stress disorder. Adolescents with SUD and psychopathological problems. Reduces consumption and improves family relationships. Adolescents with alcohol consumption and suicidal tendencies.
**Contingency** **management**	Promising. Useful BTSAS program.	Severe dual mental disorder. Severe mental disorder and stimulant use.
**Dialectical behavior therapy**	There is little evidence.	Dual personality disorders (particularly borderline). Dual eating disorders.
**Exposure therapy**	Possible effectiveness in relapses and cravings for alcohol, cannabis, methamphetamines, and opiates.	Dual anxiety disorders. Dual post-traumatic stress disorder.
**Family therapy**	Probable effectiveness.	
**Group therapy**	Effective.	Dual alcohol use disorder. Severe dual mental disorder.
**Interpersonal therapy**	Limited evidence.	
**Mindfulness**	Promising.	Relapse prevention.
**Motivational** **interviewing**	Effective, the most supported by evidence. Better results than psychoeducation.	Mental disorders and alcohol consumption. Severe dual mental disorder.
**Psychoeducation**	Useful.	
**Relapse prevention**	Effective.	Dual post-traumatic stress disorder.
**Multicomponent** **therapies**	The most effective combination is motivational interviewing and CBT. Other combinations recommended: mindfulness, psychoeducation, positive reinforcement, motivational reinforcement, social skills, and group therapy. Relaxation and grounding techniques may be useful.	Severe dual mental disorder: combination of motivational interviewing, CBT, and family interventions.

Note: Prepared by the authors based on [1,4,12,14,17,22,25,27,29,34,37,38,41,42,44,45,48]. BTSAS: Behavioral Treatment for Substance Abuse in Severe and Persistent Mental Illness [42]. CBT: cognitive behavioral therapy, SUD: substance use disorder.

### 3.3. Recommendations for the Psychological Treatment of Severe Dual Mental Disorders

This section presents the evidence-based data collected to date. When research on a specific comorbid disorder is unavailable, the most effective treatment for each disorder is recommended [14].

#### 3.3.1. Schizophrenia Spectrum and Other Psychotic Disorders

The summary of evidence and recommendations can be seen in Table 2.

- Substance-related disorders

Psychotherapy is an indispensable complement to pharmacotherapy in individual, group, and family settings. It should be adapted to each patient and their stage of treatment [49]. There is no single approach to treating dual psychosis, and it may be necessary to combine different therapeutic approaches for each patient; therefore, therapist flexibility is essential [14]. There is limited evidence that specific interventions affect different outcome parameters in patients with schizophrenia and SUDs (level of evidence C) [50]. Several studies have examined the effectiveness of CBT for psychosis symptoms and substance use, but the evidence is contradictory [14]. For example, CBT combined with motivational interventions has been shown to improve functioning and substance use, but with mixed results regarding the duration of improvement [49].

Motivational interventions facilitate a high rate of treatment compliance, promote patient participation in treatment, increase adherence and retention, and foster change [49], but adaptations may be necessary for patients with psychotic disorders and SUDs [14].

Traditional self-help groups (non-professional and non-directive) based on the twelve-step techniques, such as Alcoholics Anonymous and Narcotics Anonymous, are not suitable for people with dual psychotic disorders due to their confrontational style. Furthermore, their moralistic approach does not facilitate adequate adherence to pharmacological treatment [51].

FT can complement other treatments. A meta-analysis concluded that FT improves abstinence from substance use, although its effects on psychotic symptoms were not examined [14]. FT has also been shown to be useful in treating both disorders separately. However, no study has yet evaluated FT in the context of dual psychosis [14], and few studies exist with dynamic therapies in dual schizophrenia. More research on family interventions in patients with dual psychosis is needed to draw any conclusions [49].

Given the results of interventions targeting a single disorder, integrated treatments appear to produce more positive results and are the recommended approach for treating dual psychosis [4,14]. Integrated care for both disorders, including pharmacotherapy, MI, CBT, lifestyle interventions, case management, and interventions with family members, significantly improves both positive psychotic symptoms and substance use [14,31]. Furthermore, adapting techniques to the specific characteristics of this population may be necessary.

MI has been adapted for patients with dual psychosis, but randomized studies present contradictory results, probably due to methodological limitations and shortcomings [52]. Nevertheless, MI reduces substance use in patients with early psychosis [33]. It has also been applied to treat dual psychosis in adults and adolescents, proving especially effective in patients who are less motivated to change and have greater difficulty adhering to treatment [51]. Dual disorder self-help groups (Double Trouble in Recovery), which focus on the specific problems of individuals with dual diagnosis, have been implemented; however, research on this topic is lacking [21]. Among DD patients, those with schizophrenia seem to benefit the least from these groups. Only a minority engage in self-help approaches, likely due to deficits in social skills [49]. Self-help groups that use an educational methodology integrating cognitive elements have also been used, but their effectiveness has not been established [51].

Modified CBT [53], which includes RP, MI, and CM strategies in a group format that considers cognitive limitations, has been used to treat DDs [21]. Modified Motivation Enhancement Therapy [54] has also been used to address the cognitive challenges and reasons for low motivation to stop substance use in patients with psychosis [21]. Another therapy used for dual psychosis is Dual Recovery Therapy [55], which integrates RP, motivation enhancement therapy, the principles of the twelve-step program, and social skills training for psychiatric disorders.

- Alcohol-related disorders

The limited number of studies conducted allows us to recommend motivational and cognitive behavioral interventions to reduce alcohol consumption (weak recommendation [56], level of evidence B [50]). Meta-analyses and systematic reviews report the efficacy of MI, CBT, and MI combined with CBT, CM, and GT [57].

MI has been adapted to treat individuals with schizophrenia who consume alcohol in a twelve-session treatment package, with positive results in reducing alcohol consumption and increasing abstinence compared to PE [49]. Better abstinence rates at six months have also been found with MI compared to usual care [19]. In summary, patients with psychosis and comorbid alcohol use disorders should receive psychotherapy after adequate stabilization, with MI, CBT, GT if possible, and CM recommended [57].

- Cannabis-related disorders

Psychological interventions, such as CBT and MI, show promise and effectiveness in the short term, while more extensive interventions are recommended for heavy users with chronic disorders [33]. Studies have shown that CM is effective in promoting abstinence [14]. PE and CBT programs have shown promise in reducing cannabis use in patients with a first episode of psychosis, and brief interventions should be implemented to prevent cannabis use among individuals with psychosis, even if they are using small amounts [31].

Currently, CBT is the most widely used treatment for cannabis use in the absence of other proven effective treatment options [31], although there is no evidence indicating the superiority of specific interventions [50].

- Inhalant-related disorders

These DDs should be treated with standard CBT approaches, such as assertiveness and coping skills and alternatives to substance use, supplemented with community reinforcement, family interventions, and an assertive approach [31].

Disorders related to sedatives, hypnotics or anxiolytics

There is no evidence of any specific treatment [58].

- Stimulant-related disorders

Better results in abstaining from stimulants have been found at twelve weeks with CM compared to usual treatment [19]. For cocaine-related disorders, CBT, CM, RP, MI, and self-help groups have demonstrated effectiveness [59]. Programs with CM have demonstrated a reduction in consumption [49].

- Tobacco-related disorders

Although the data are inconclusive, specific psychosocial interventions are recommended for patients with schizophrenia and comorbid smoking (level of evidence C) [50], as there is supporting evidence of their benefits. Thus, a brief motivational intervention is recommended for patients in the precontemplation or contemplation stage, followed by a reassessment of the change stage at a later time, while psychological counseling is suggested in the preparatory or action stages [60]. Motivational counseling, as a complement to other interventions to achieve abstinence and reduce consumption, is effective [34], as are group treatment programs including peer support [61]. For patients with schizophrenia, reducing cigarette consumption can be a useful intermediate goal initially, as opposed to abrupt cessation [62]. Furthermore, it is considered good clinical practice to initiate smoking cessation only in patients with stable psychopathology [50].

- Gambling disorder

CBT, especially when combined with MI and self-help groups, appears to be effective at treating gambling disorder [49,63]. According to the clinical guideline by Fernández-Artamendi et al. [63], CBT was effective in achieving abstinence from gambling, reducing gambling episodes, and decreasing money spent on gambling during follow-up compared to the control group. Additionally, combining CBT with third-generation strategies may also be effective for these patients, though more research is needed in this area.

**Table 2:** Recommendations for the psychological treatment of schizophrenia and other dual psychotic disorders.

Schizophrenia Spectrum and Other Psychotic Disorders		
Substance Use Disorder	Recommended Therapies	Specific Recommendations
**Substance-related** **disorders**	Individual, group and family psychotherapy are an indispensable complement to pharmacotherapy. Integrated treatment approach recommended, including pharmacotherapy, motivational interviewing, CBT, lifestyle interventions, case management, and interventions with family members. Motivational interviewing, CBT (individual and group), psychoeducation, relapse prevention, contingency management, group therapy and family therapy may be effective. Recommended combination: motivational interviewing and CBT.	It may be necessary to adapt the techniques to the characteristics of these dual pathologies. Motivational interviewing reduces substance use in patients with early psychosis. Motivational interviewing is more effective in patients less motivated to change and who have more difficulty with therapeutic compliance. Motivational interviewing leads to a greater reduction in alcohol consumption compared to standard care. Classical twelve-step groups are not suitable for individuals with dual psychosis.
**Alcohol-related** **disorders**	Motivational and cognitive behavioral interventions are effective. CBT, motivational interviewing alone or combined with CBT, contingency management, and group therapy are effective.	Motivational interviewing leads to a greater reduction in alcohol consumption compared to standard care.
**Cannabis-related** **disorders**	There is no evidence for specific interventions. CBT and motivational interviewing are the most promising. Contingency management effective to promotes abstinence.	For heavy users with chronic disorders, more extensive interventions are recommended. For patients with a first episode of psychosis, psychoeducation and cognitive behavioral therapy show promise. Brief interventions are recommended for the prevention of psychosis among individuals who may be consuming even small amounts.
**Inhalant-related** **disorders**	CBT with community reinforcement, family interventions and assertive approach.	
**Disorders related to sedatives, hypnotics or anxiolytics**	There is no evidence.	
**Stimulant-related** **disorders**	Contingency management leads to better abstinence outcomes compared to usual treatment.	
**Cocaine-related** **disorders**	CBT, contingency management, relapse prevention, motivational interviewing, and self-help groups are effective. Contingency management leads to reduced consumption.	
**Tobacco-related** **disorders**	Cognitive and psychosocial interventions should be offered to all patients (reduction or cessation). Contingency management is an effective complement (reduction and abstinence). Group treatment programs, including peer therapy, are effective.	Brief motivational interventions in pre-contemplative or contemplative stage. Psychological counseling in preparatory or action stage. Reducing consumption can initially serve as a useful intermediate goal. Considered a good clinical practice to initiate smoking cessation only in patients with stable psychopathology.
**Gambling disorder**	Effectiveness of CBT, especially combined with motivational interviewing and self-help groups.	

Note: Prepared by the authors based on [14,17,19,21,24,26,31,33,34,42,49,50,51,54,56,57,58,59,60,61,62,63]. CBT: cognitive behavioral therapy.

#### 3.3.2. Schizoaffective Disorder

Adding CM to treatment improves the evolution of SUD and psychiatric symptoms, thereby decreasing the likelihood of hospitalization [59,64].

#### 3.3.3. Bipolar Disorder and Related Disorders

The summary of evidence and recommendations can be seen in Table 3.

- Substance-related disorders

Although limited research has examined non-pharmacological approaches to comorbid BD and SUDs management [14], group CBT, integrated therapy, and RP techniques appear to help reduce hospitalizations, increase abstinence, improve medication adherence, reduce addiction severity, and, to a lesser extent, improve mood symptoms. However, results are inconsistent across studies, highlighting the need for further research [4].

Integrated group therapy (IGT), which is specifically developed for patients with BD and SUD, focuses on RP strategies and is manualized [65]. It is the most well-researched intervention for this comorbidity to date [14]. IGT has been associated with greater abstinence, fewer days of substance abuse, and fewer days of drinking alcohol to intoxication than treatment as usual [4]. A non-randomized pilot study and two small randomized clinical trials have shown more positive findings regarding alcohol and other drug use outcomes compared to the GT control conditions, but not regarding mood [14]. Although these results are promising, further research is needed [19].

Another therapy specifically designed for patients with BD is Interpersonal and Social Rhythms Therapy [66]. There is evidence of its effectiveness in promoting well-being, improving interpersonal functioning and life satisfaction, and stabilizing daily activity schedules and social and interpersonal relationships has a beneficial effect, even on SUD [21]. This therapy was adapted to create Social Rhythm Therapy [67] to facilitate its implementation in routine clinical practice.

Psychological GTs with PE, RP, and family involvement have proven beneficial in reducing symptoms, promoting abstinence, and improving treatment adherence [20]. Furthermore, brief MI integrated into the usual treatment of hospitalized patients with dual BD improved treatment adherence at three months [49].

According to the review by Secades-Álvarez and Fernández-Rodríguez [20], community-based GT was also effective in reducing alcohol and stimulant use and increasing improvement in manic and depressive symptoms. The review concludes that integrated community-friendly GT is more effective than drug counseling groups when comparing the different interventions. Thus, it reduces substance use and psychiatric symptoms with a better overall clinical outcome, although in both cases there is apparent efficacy in addressing DDs. Furthermore, family intervention for dual diagnosis proved more beneficial in education about disorders, psychiatric symptoms, and the severity of substance use than family PE.

In summary, IGT is recommended (weak recommendation) for reducing substance use in these DDs [19].

- Alcohol-related disorders

IGT should be highlighted in addition to the existing treatment options for addiction [56].

- Stimulant-related disorders

A study has evaluated CM with promising results [64]. For cocaine-related disorders, adding CM to the treatment improves the evolution of SUD and psychiatric symptoms, decreasing the likelihood of hospitalization [59].

#### 3.3.4. Depressive Disorders

Table 4 shows the summary of evidence and recommendations.

- Substance-related disorders

Integrated psychological treatment for depression and SUD is a promising approach, but there is insufficient empirical support for its effectiveness in treating depression (weak recommendation) [19]. There are few studies on the treatment of dual depression, and most of these have been conducted on patients with alcohol dependence [68]. Although research increasingly supports the use of integrated psychological treatments for dual depression, clinical trials are needed to overcome current methodological limitations, small sample sizes, and heterogeneity in results [14].

CBT is effective for treating dual depression both as a standalone treatment and in combination with pharmacological treatment, although it appears that some of this improvement is lost upon completion of treatment (level of evidence 2) [17,22,25]. Adding CBT and MI to the standard therapeutic approach is also beneficial [69]. Integrated CBT and group CBT approaches, with or without the complementary use of antidepressants, can reduce substance use and depressive symptoms and improve short- and long-term functioning [4]. Reviews and meta-analyses have shown that integrated CBT approaches provide superior results for depression and substance use compared to treatment as usual or no treatment. However, there is currently insufficient evidence to conclude that one psychological therapy is more effective than another [14].

Third-generation CBTs, such as ACT or interventions based on MF and BA, have fewer studies regarding these DDs, although initial findings are promising [70]. Integrating behavioral support into CBT treatment is a viable and effective way to reduce depressive symptoms, increase activation of pleasurable behaviors, and improve treatment retention [4]. Furthermore, it effectively reduces the quantity and frequency of substance use [14].

The BTSAS behavioral treatment program [42] has been used with promising results compared to supportive therapy [49].

In the Building Recovery by Improving Goals, Habits, and Thoughts [71] project, the effectiveness of residential treatment for SUD was compared to residential treatment with added CBT. The best results were observed in patients who received CBT, with greater adherence to treatment and improvement in depressive symptoms [68,72]. Providing group CBT for depression to patients in residential treatment for SUD presents better cost-effectiveness and cost-utility ratios than other interventions [73].

In summary, CBT is the most well-researched psychotherapy, and the results establish that it can be considered the most effective, among those available, in the treatment of comorbid SUD and depressive disorders [70]. The use of integrated therapeutic approaches with CBT is recommended, especially in combination with additional treatment strategies such as RP therapy or MI [4].

- Alcohol-related disorders

Therapies that have demonstrated efficacy in reducing alcohol consumption in these dual-diagnosis patients include motivational therapy, CBT, RP therapy, CM, and the twelve-step model [56]. CBT is associated with a decrease in consumption after treatment [31]. A combination of MI and CBT achieves a greater reduction in consumption than brief therapy alone, with the computerized version showing a superior effect compared to the traditional therapist-led intervention [7].

A systematic review with meta-analysis, conducted over a decade ago, evaluated the effectiveness of CBT and motivational interventions compared to usual care [74]. The review concluded that both interventions produced clinically significant improvements in reducing depressive symptoms and alcohol consumption. Subsequent results have confirmed this finding, although the effect size is small [19] and lower than that obtained with pharmacological treatments [68]. Usual therapy supplemented with CBT and MI provides small but significant effects in improving major depression and reducing alcohol consumption, compared with usual therapy alone or other brief psychosocial interventions [4]. However, evidence has shown that short-term interventions in patients with major depression and alcohol use disorder may be ineffective; therefore, the use of cognitive therapy, behavioral therapy, and supportive therapy is recommended, as there is insufficient evidence for other forms of therapy [57].

In summary, most available studies have reported that CBT is effective in treating patients with comorbid depression and alcohol use disorders, improving both depressive symptoms and alcohol consumption [57]. Therefore, CBT is recommended (weak recommendation) to reduce alcohol consumption and the level of internalization (depression and anxiety) in patients with these DDs [19].

- Cannabis-related disorders

As Arias et al. [33] pointed out in their clinical guideline, psychological interventions are promising and effective in the short term. Two recommendations stand out: CBT and MI have the best results, and in heavy users with chronic cannabis use disorder, interventions should be more extensive. They also state that combining MI and CBT achieves a greater reduction in consumption than brief therapy alone, and that the computerized version has a superior effect than traditional therapist-led intervention.

In summary, CBT is currently the most widely used and recommended treatment for comorbid depressive disorders and cannabis use [31], although more recent studies are needed to refine or consolidate the recommendations in this area, as there has been a change in the type of substance consumed under the name of cannabis.

- Inhalant-related disorders

These DDs should be treated with standard CBT approaches (assertiveness and coping skills, and alternatives to substance use), which should be complemented by community reinforcement, family interventions and an assertive approach [31].

- Opioid-related disorders

Studies have shown that CBT is effective in promoting opioid abstinence among individuals with concurrent major depression who are receiving buprenorphine maintenance therapy [14]. Similarly, neurofeedback training for opioid addiction has demonstrated improvements in craving and depression [75]. Furthermore, CBT provides an additional benefit when combined with a maintenance therapy program for treating depression in opioid users [31].

- Disorders related to sedatives, hypnotics or anxiolytics

Psychological and behavioral therapy can effectively treat insomnia, and CBT can effectively treat depression, potentially being more effective if sedation and anxiolysis are minimal due to benzodiazepine use [31]. However, there is no evidence of a specific treatment for this comorbidity [58].

- Stimulant-related disorders

CBT can address stimulant use, including methamphetamine, and has been shown to be effective [31]. CM has also been evaluated in a previous study, with promising results [64].

In amphetamine-related disorders, psychological approaches alone have not demonstrated sufficient effectiveness nor have they demonstrated superior effectiveness in combination with pharmacological treatments. As Hellem et al. [76] point out in their review, there is a significant knowledge gap regarding the treatment of methamphetamine use disorder and comorbid depression. Furthermore, since female amphetamine users experience higher rates of depressive disorders than men do, a gender-specific treatment approach is necessary.

Cognitive and/or behavioral therapy interventions have shown utility in cocaine-related disorders, and adding CM to treatment improves the course of SUDs and psychiatric symptoms, decreasing the likelihood of hospitalization [59]. Additionally, CM has been shown to promote cocaine abstinence among individuals with comorbid major depression [14].

- Tobacco-related disorders

As reviewed by Morozova et al. [77], adding behavioral components to pharmacological treatment is a promising strategy for smokers with depressive symptoms, as reviewed by Morozova et al. [77]. The researchers concluded that incorporating a psychosocial mood management component into standard smoking cessation interventions increases abstinence rates in smokers with current or past major depression compared to standard treatment alone. Furthermore, including this component has a beneficial, albeit small, effect on the likelihood of quitting smoking. CM, when used alongside other interventions to achieve abstinence or reduce consumption, is also effective in people with depression [34].

- Behavioral addictions

Regarding gambling disorder, CBT, especially in combination with MI and self-help groups, appears to be effective in these DDs, as reviewed by Fernández-Artamendi et al. [63] in their guide. Thus, there are studies that point to the effectiveness of CBT, or even a brief telephone intervention, in alleviating symptoms of depression and gambling problems.

Regarding internet addiction, several studies indicate that CBT and other psychosocial interventions can effectively reduce online time and the presence of depressive symptoms [78]. Further research is needed to update and improve our understanding of the use of psychotherapy in treating this disorder, given the increasing demand for treatment among young people.

**Table 4:** Recommendations for the psychological treatment of dual depressive disorders.

Depressive Disorders		
Substance Use Disorder	Recommended Therapies	Specific Recommendations
**Substance-related** **disorders**	There are few studies and most of them focus on patients with alcohol dependence. Integrated treatment shows promise, but there is a lack of empirical support for improving depression. CBT, alone or combined with psychopharmacological treatment, is the most effective treatment available. Adding CBT and motivational interviewing to standard treatment is beneficial. Integrated CBT or group CBT, with or without antidepressants, has shown better results than usual treatment or no treatment. Acceptance and commitment therapy, mindfulness, behavioral activation, and BTSAS show promise. Integrated approaches combining CBT, relapse prevention, and motivational interviewing are recommended.	Adding CBT to residential treatment for SUD improves outcomes and has better cost-effectiveness and cost–utility ratios than other interventions.
**Alcohol-related** **disorders**	Efficacy of motivational therapy, CBT, relapse prevention, contingency management, and the twelve-step model. CBT, cognitive therapy, behavioral therapy, and supportive therapy are recommended. Motivational interviewing and combined CBT resulted in a greater reduction in consumption than brief therapy. Usual treatment with CBT and motivational interviewing yields better results than only usual or other interventions.	
**Cannabis-related** **disorders**	CBT, the most recommended, and motivational interviewing are the most promising. The combination of CBT and motivational interviewing reduces consumption more than brief therapy.	More extensive interventions are recommended for heavy users with chronic disorders.
**Inhalant-related** **disorders**	CBT with community reinforcement, family interventions and assertive approach.	
**Opioid-related** **disorders**	Neurofeedback training can reduce cravings and depression. CBT provides an additional benefit combined with a maintenance therapy program.	Effective contingency management to promote abstinence in people receiving buprenorphine maintenance treatment.
**Disorders related to sedatives, hypnotics or anxiolytics**	There is no evidence regarding any specific treatment. Psychological and behavioral treatment can be effective for insomnia. CBT is more effective if sedation and anxiolysis are minimal due to the use of benzodiazepines.	
**Stimulant-related** **disorders**	Effective CBT to address substance use. Promising contingency management.	
**Amphetamine-related disorders**	Evidence is lacking.	A gender-specific treatment approach is needed, since women experience higher rates of depression than men.
**Cocaine-related** **disorders**	Useful cognitive and/or behavioral interventions. Adding contingency management improves the course and abstinence of SUD and psychiatric symptoms.	
**Tobacco-related** **disorders**	Adding behavioral components to pharmacological treatment is a promising strategy. Adding a psychosocial mood management component to the standard intervention increases abstinence rates. Contingency management is effective as a complement to other interventions in abstinence and consumption reduction.	
**Gambling disorder**	CBT, particularly combined with motivational interviewing and self-help groups, appears to be effective. Effectiveness of CBT or brief telephone intervention to improve depressive symptoms.	
**Internet addiction**	CBT and other psychosocial interventions can be effective in reducing online time and depressive symptoms.	

Note: Prepared by the authors based on [4,7,14,17,22,31,34,42,49,56,57,58,59,63,64,68,69,70,73,76,77,78]. BTSAS: Behavioral Treatment for Substance Abuse in Severe and Persistent Mental Illness [42]. CBT: cognitive behavioral therapy; SUD: substance use disorder.

#### 3.3.5. Obsessive Compulsive Disorder (OCD) and Related Disorders

- Substance-related disorders

There is limited evidence on the treatment of OCD in people with SUDs, as most studies on OCD either exclude substance use [14] or do not assess or mention it. One randomized clinical trial has examined the efficacy of CBT with exposure and response prevention (ERP) for treating dual OCD among individuals undergoing residential substance use rehabilitation. The trial revealed that patients who received CBT with ERP remained in treatment longer, had less severe OCD symptoms, and higher abstinence rates during treatment and at the twelve-month follow-up, compared to patients who received treatment for substance use only or for substance use plus progressive muscle relaxation [14].

While these findings are promising, more research is needed to gather evidence, not only on ERP but on other psychotherapeutic approaches, since the relationship between OCD and substance use suggests the need to develop integrated treatments that simultaneously address both disorders: PE, substance use, ERP, and therapeutic work focused on increasing self-efficacy, to help the patient believe that they can cope with the situation without substance use [14].

- Alcohol-related disorders

There is no evidence supporting a recommendation [56].

## 4. Discussion

This consensus has several limitations. No quantitative measures were used to reach consensus, which may limit reliability. Most of the available evidence on the psychological treatment of dual diagnosis is level 3 or 4, and most recommendations are grade D or weak. Throughout the text, there may be inconsistencies in the use of the terms DD, SMD, comorbidity, and integrated treatment because we have tried to use the original terms used by different authors. This limitation is common in this field of study and reflects the need for standardization of the terms used [2]. It is also important to note that all meetings and the selection of participating experts were conducted within WADD/SEPD conferences, so this selection bias may limit the generalizability of the results. The same applies to geographical origin. Finally, since this is not a systematic review, the PRISMA diagram, which would further explain the procedure, has not been created.

These limitations indicate that the results should be interpreted with caution until higher-quality research on this topic is available. However, we believe that this consensus, together with the concise summary of the tables, can be a useful reference tool in daily clinical practice and stimulate the production of new knowledge. Given the heterogeneity of results, clinicians are advised to follow the recommendations with the highest level of evidence and, in the absence of such evidence, to use therapies that have shown efficacy in both disorders separately.

The recommendations for future research are as follows:

- Randomized clinical trials comparing different psychological treatments for each of the severe mental disorders;
- Specify the diagnoses of the participating subjects, including their specific SUD;
- Standardize diagnostic criteria and interventions used;
- Use mixed methods that combine quantitative and qualitative data;
- Improve the quality of research on psychological treatment, especially in areas with less available evidence, such as obsessive compulsive disorder and schizoaffective disorder;
- Improve the quality of research in understudied populations, such as children and adolescents, the elderly, the prison, the homeless or the diverse sexual and gender populations;
- Design and evaluate gender-specific interventions;
- Design and evaluate eHealth interventions;
- Study more complex comorbidities (e.g., SUDs plus two SMDs) in order to design and evaluate integrated tailored interventions [79];
- Evaluate the factors common to psychotherapies and the transdiagnostic mechanisms of their effectiveness, for example, self-regulation, emotional regulation, motivation for change, metacognition, and social functioning. Evidence is lacking due to the absence of studies in populations with DDs and the exclusion of subjects with SUDs from the studies [80].
- Establish research and funding priorities to optimize efforts and resources. Recommended areas for priority include obsessive compulsive disorder, schizoaffective disorder, adolescents, gender, e-Health, and common factors in psychotherapies.
- Study the implementation challenges of therapies, examining the practical barriers: lack of training of professionals [81], lack of specific resources [82], and persistence of the parallel treatment model [68], with the consequent fragmentation of services [12], duplication of resources and professionals [83], and “the wrong door syndrome” [4].

## 5. Conclusions

The main conclusions of this first part of the consensus are as follows:

1. Psychological treatment appears to be effective in the therapeutic approach to dual SMDs compared to stand-alone or parallel treatment models.
2. Integrated psychological treatment appears to be more effective than other treatment models for dual SMDs.
3. The psychological interventions with the most empirical evidence for treating dual SMDs are MI, CBT, and RP. The strongest evidence supports combining MI and CBT, which often includes RP. Family interventions are also useful.
4. Multicomponent strategies are proposed as the best alternative for addressing all aspects required for intervention in patients with DDs, with a proactive approach adapted to each stage, despite the impossibility of associating efficacy with a specific component.
5. The level of evidence available on psychological treatment varies considerably among different specific comorbidities. The most well-studied MDs are those within the schizophrenia spectrum and depressive disorders.
6. In the treatment of dual schizophrenia, MI, integrated CBT and its combination with MI, PE, RP, CM, brief interventions based on MI principles and more oriented towards counseling, and family interventions, have shown efficacy with low to moderate evidence and should be used if available.
7. For dual depressive disorders, CBT is the most researched psychotherapy with the most well-established results. Integrated therapeutic approaches combining CBT with additional strategies such as RP or MI are recommended.
8. For dual BD, group psychological therapies with PE or RP and family involvement, CM, and family intervention have shown positive results, although there is very little evidence. Social rhythm therapy improves interpersonal functioning and is effective in RP.
9. More empirical evidence is needed on the psychological treatment of dual SMDs, particularly for OCD and schizoaffective disorder.
10. More randomized controlled clinical trials are needed to provide robust effectiveness data and overcome methodological limitations, mainly lack of randomization and control, small sample sizes, and heterogeneity in study variables and results.

## Figures and Tables

**Table 3:** Recommendations for the psychological treatment of dual bipolar disorder.

Bipolar Disorder		
Substance Use Disorder	Recommended Therapies	Specific Recommendations
**Substance-related** **disorders**	Group CBT, integrated group therapy (most recommended), and relapse prevention are effective, but more research is needed. Probable effectiveness of group psychological therapies with psychoeducation, contingency management, relapse prevention and family inclusion. Community-friendly integrated group therapy is more effective than drug counseling groups.	Interpersonal and Social Rhythms Therapy for relapse prevention and improvement of interpersonal functioning and life satisfaction. Brief motivational interviews integrated into the usual treatment to improve adherence to treatment. Family intervention for dual disorders is more beneficial than family education.
**Alcohol-related** **disorders**	Integrated group therapy.	
**Stimulant-related** **disorders**	Promising contingency management.	
**Cocaine-related** **disorders**	Adding contingency management to treatment improves the course of SUD and psychiatric symptoms.	

Note: Prepared by the authors based on [4,14,19,20,49,56,59,64,65,66]. CBT: cognitive behavioral therapy, SUD: substance use disorder.

## Data Availability

No new data were created.

## Abbreviations

The following abbreviations are used in this manuscript:

DD	Dual Disorder
SUD	Substance Use Disorder
MD	Mental Disorder
SMD	Severe Mental Disorder
BD	Bipolar Disorder
APD	Antisocial Personality Disorder
BPD	Borderline Personality Disorder
PTSD	Posttraumatic Stress Disorder
SEPD	Spanish Society of Dual Pathology
WADD	World Association of Dual Disorders
RP	Relapse Prevention
MI	Motivational Interviewing
ACT	Acceptance and Commitment Therapy
BA	Behavioral Activation
CBT	Cognitive Behavioral Therapy
CM	Contingency Management
BTSAS	Behavioral Treatment for Substance Abuse in Severe and Persistent Mental Illness
DBT	Dialectical Behavior Therapy
PD	Personality Disorder
FT	Family or Family-based Therapy
GT	Group Therapy
MF	Mindfulness
PE	Psychoeducation
IGT	Integrated Group Therapy
OCD	Obsessive Compulsive Disorder
ERP	Exposure and Response Prevention
